# Practical Points

**Published:** 1897-02-06

**Authors:** 


### PRACTICAL POINTS.

*The Temperature of Rooms.*—"*A Correspondent*" *asks: Is there any means (without involving heavy structural alterations) of maintaining the temperature of a room or rooms below 60 degs. in the summer time, when perhaps the temperature outdoors is 90 degs. and indoors 70 degs.? I should not mind expending up to £20 to secure the desired result.*

